# Diabetic neuropathy: Past, present, and future

**DOI:** 10.22088/cjim.14.2.153

**Published:** 2023

**Authors:** Juan Quiroz-Aldave, María Durand-Vásquez, Elman Gamarra-Osorio, Jacsel Suarez-Rojas, Pela Jantine Roseboom, Rosa Alcalá-Mendoza, Julia Coronado-Arroyo, Francisca Zavaleta-Gutiérrez, Luis Concepción-Urteaga, Marcio Concepción-Zavaleta

**Affiliations:** 1 Division of Medicine, Hospital de Apoyo Chepén, Chepén, Peru; 2 Division of Family Medicine. Hospital de Apoyo Chepén, Chepén, Peru; 3 Division of Endocrinology, Hospital Víctor Lazarte Echegaray, Trujillo, Peru; 4 Universidad Científica del Sur, Lima, Peru; 5 Division of Emergency Medicine, Hospital Regional Docente de Trujillo, Trujillo, Peru; 6 Division of Physical Medicine and Rehabilitation, Hospital Víctor Lazarte Echegaray, Trujillo, Peru; 7 Division of Obstetrics and Gynecology, Hospital Nacional Edgardo Rebagliati Martins, Lima, Peru; 8 Division of Neonatology, Hospital Belén de Trujillo, Trujillo, Peru; 9 School of Medicine, Universidad Nacional de Trujillo, Trujillo, Peru; 10 Division of Endocrinology, Hospital Jorge Voto Bernales, Lima, Peru

**Keywords:** Diabetic Neuropathy, Diabetes Mellitus, Complication, Treatment, Glycemic Control, HbA1c

## Abstract

**Background:**

A sedentary lifestyle and an unhealthy diet have considerably increased the incidence of diabetes mellitus worldwide in recent decades, which has generated a high rate of associated chronic complications.

**Methods:**

A narrative review was performed in MEDLINE, EMBASES and SciELO databases, including 162 articles.

**Results:**

Diabetic neuropathy (DN) is the most common of these complications, mainly producing two types of involvement: sensorimotor neuropathy, whose most common form is symmetric distal polyneuropathy, and autonomic neuropathies, affecting the cardiovascular, gastrointestinal, and urogenital system. Although hyperglycemia is the main metabolic alteration involved in its genesis, the presents of obesity, dyslipidemia, arterial hypertension, and smoking, play an additional role in its appearance. In the pathophysiology, three main phenomena stand out: oxidative stress, the formation of advanced glycosylation end-products, and microvasculature damage. Diagnosis is clinical, and it is recommended to use a 10 g monofilament and a 128 Hz tuning fork as screening tools. Glycemic control and non-pharmacological interventions constitute the mainstay of DN treatment, although there are currently investigations in antioxidant therapies, in addition to pain management.

**Conclusions:**

Diabetes mellitus causes damage to peripheral nerves, being the most common form of this, distal symmetric polyneuropathy. Control of glycemia and comorbidities contribute to prevent, postpone, and reduce its severity. Pharmacological interventions are intended to relieve pain.

From the ancient age in which observations on DN began until the present, there has been notable progress in the description of this pathology and the understanding of its underlying pathological mechanisms and treatments. Based on Skljarevski (1) and Boulton (2) Figure 1 depicts diabetic neuropathy historically, up to the present. Last decades, the acquisition of inadequate lifestyles has caused a large increase worldwide in components of the metabolic syndrome, including diabetes mellitus (DM) (3–5). The combination of genetic susceptibility and other factors such as a sedentary lifestyle and overeating are responsible for the appearance of DM. Prevention of the development of DM and its complications is essential to reduce the high morbidity and mortality it causes (4, 6, 7). Neuropathy as a complication of DM is associated with large social and health costs, in addition to a decrease in the quality of life (8–11). About half of the cases of neuropathy are secondary to DM (12). Diabetic neuropathy (DN) affects 30-50% of people with DM. The prevalence of DN in newly diagnosed diabetics is 8%, reaching more than 50% in those with long-standing DM (10, 13-16). 

It is interesting that, although the incidence of neuropathy in people with type 2 DM (T2DM) is higher than in those with type 1 DM (T1DM), its prevalence is similar, which is probably due to differences in age of onset and its pathophysiology (17–22). DM is the main risk factor involved in the genesis of neuropathy, and its main predictors are the duration of DM and hemoglobin A1C (HbA1c) levels (22). Several epidemiological studies have shown that obesity is the second most important risk factor for the development of DN (23–29). The other components of the metabolic syndrome (hypertriglyceridemia, hypertension, and low levels of high-density lipoproteins) are also associated with DN in patients with T2DM and in some patients with T1DM (24, 25, 30). Other risk factors involved are smoking, alcoholism, tall stature, and advanced age (31). The role of genetics in the development of DN is not yet fully understood, so more research is required for future applications (32). The goal of this review is to describe the pathophysiology, clinical manifestations, diagnosis, and treatment of DN, and mention the current advances and future perspectives on its management.

**Figure 1:**
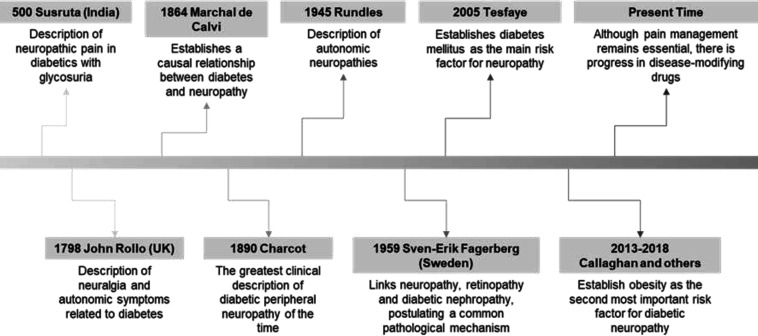
Diabetic neuropathy research timeline

## Methods

**Search strategy**: MEDLINE, EMBASES and SciELO Electronic databases were searched for completed studies of any design except case reports, case series, letters to the editor, and conference proceedings from database inception between 2005 and 2022. The Medical Subject Heading (MeSH) used was "diabetic neuropathies" and includes the following concepts: diabetic autonomic neuropathy, diabetic neuralgia, symmetric diabetic proximal motor neuropathy, asymmetric diabetic proximal motor neuropathy, diabetic asymmetric polyneuropathy, diabetic mononeuropathy, diabetic amyotrophy, and diabetic polyneuropathy. 

**Inclusion and exclusion criteria**: Inclusion criteria were studies published in English or Spanish, involving patients of any age. Systematic reviews, clinical trials, prospective cohort studies, cross-sectional and retrospective studies, and narrative reviews related to the objective of this manuscript were included. The investigation was limited to articles related to human beings, so that exclusion criteria were non-human studies. Case reports, case series, letters to the editor and conference proceedings were also excluded.

**Figure 2:**
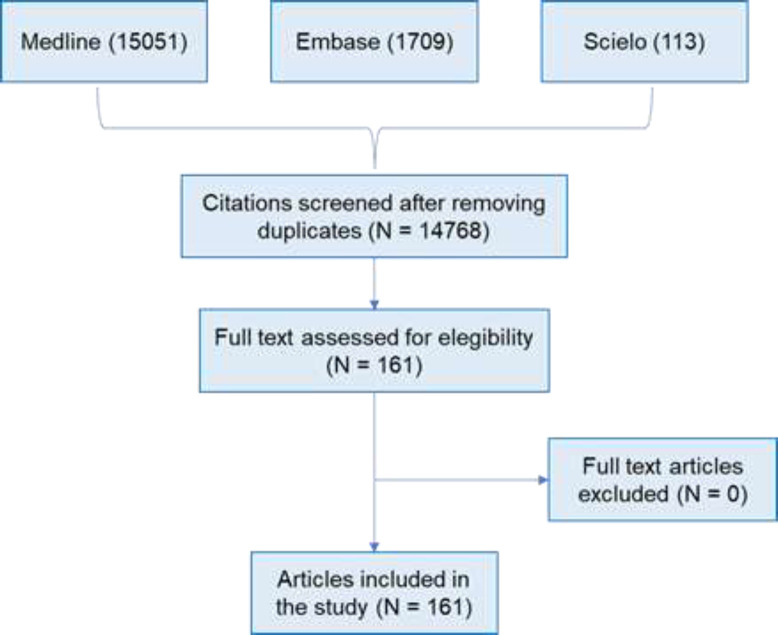
Flowchart of narrative review process

**Screening**: Identified citations were exported to Endnote. A total of 16873 citations were identified; 2105 duplicates were removed; 14768 titles and abstracts were screened against eligibility criteria. No grey literature was included. 14607 titles were excluded at the title and abstract screen, 162 eligible full text papers met the inclusion criteria. This process is summarized in Figure 2 Data extraction and synthesis: Results from the included papers were extracted to a table and include physiopathology, diagnosis, management, and therapies under study.

**Quality assessment:** The quality of our narrative review was evaluated using the SANRA scale, which included the following items: explanation of the importance of the review, statement of the objectives of the review, description of the bibliographic search, references, scientific reasoning, and appropriate presentation of information (33).

This narrative review has the endorsement of the Ethics Committee of the Faculty of Medicine of the National University of Trujillo.

## Results

**Physiopathology**

DN is a degenerative problem of the nervous system that mainly affects sensory and autonomic axons, and progressively, to a lesser degree, motor axons. The exact mechanism by which DM targets sensory neurons remains unknown. DN develops progressively and involves the retraction and death of terminal sensory axons in the periphery, with relative preservation of the soma. Initially, the longest sensory axons are damaged, so the manifestations are first distal, developing to proximal. For this reason, DN is considered to be a length-dependent neuropathy (34). Chronic hyperglycemia causes damage to the Schwann cells, leading to demyelination in the most severe cases of DN. Given the interaction and mutual support between the axons and the Schwann cells, the damage of the latter would also lead to various alterations in the axons (35, 36).

**Role of hyperglycemia and hyperlipidemia**

The difference in the way of energy production in the peripheral nervous system of people with DM is the basis for understanding the pathogenesis of DN. When long-chain fatty acids are transported to Schwann cells and their axons in the dorsal root ganglia (DRG) to undergo β-oxidation, one molecule of acetyl-CoA is formed in each cycle, and it enters the tricarboxylic acid cycle for the formation of reduced nicotinamide adenine dinucleotide (NADH) and reduced flavin adenine dinucleotide (FADH2). DM has an overload of substrates that saturate the transport system, converting acetyl-CoA molecules into acylcarnitine. Accumulated acylcarnitine which is toxic to the Schwann cells and DRG neurons, is released from Schwann cells and can induce axonal degeneration, by mitochondrial dysfunction and maladaptation to stress, adding the nervous system injury (37, 38).

NADH and FADH2 enter the mitochondria by complexes I-IV to produce ATP through oxidative phosphorylation. This process generates reactive oxygen species (ROS) as by-products, but in small quantities, which are easily neutralized by cellular antioxidants such as superoxide dismutase, glutathione, and catalase (39, 40). Excessive substrate in DM leads to oxidative phosphorylation failure, decreased ATP production, and increased ROS levels, producing mitochondrial failure and metabolic and oxidative damage to Schwann cells and neurons. Of the DRGs (41–43). Dysfunctional mitochondria do not produce enough energy and lose their normal capacity for transit through the axons, worsening axonal disruption and damage (44). These alternate routes to process the excess of glucose, as the polyol pathway and the hexosamine pathway, produce an excess of free fatty acids catabolized by β-oxidation causing damage to Schwann cells, through ROS and inflammation produced by the activation of macrophages and subsequent production of cytokines and chemokines. (45–47) Additionally, hyperglycemia leads to the glycation of structural and functional proteins, generating advanced glycation end-products (APGPs), which alter or decrease protein function and interact with specific PFGA receptors, modifying gene expression and intracellular signaling. (34) They also promote the release of pro-inflammatory molecules and free radicals. (48) Furthermore, in neurons, excess cholesterol is oxidized to oxysterols, which cause tissue damage (40, 49–51). Plasma lipoproteins, mainly low-density lipoproteins (LDL), are oxidized by ROS and bind to oxidized LDL receptor 1 (LOX1), toll-like receptor 4 (TLR4) and PFGA (52–54). The binding of oxidized LDL to these receptors activates a series of signaling cascades, including the activation of caspase 3 and the degradation of nuclear DNA, producing additional inflammation and the accumulation of ROS, thus contributing to progression of neuropathy (54, 55). Sphingolipid metabolism in people with T2DM is also altered, resulting in the formation of atypical deoxysphingolipids, which are toxic to neurons and pancreatic beta cells (56, 57). The level of deoxysphingolipids not only is elevated in people with metabolic syndrome or T1DM but much more in those with T2DM. Of the latter, those with DN present the highest levels (58, 59). Excess of glucose and fatty acids and their relation to inflammation and tissue damage.

**Figure 3:**
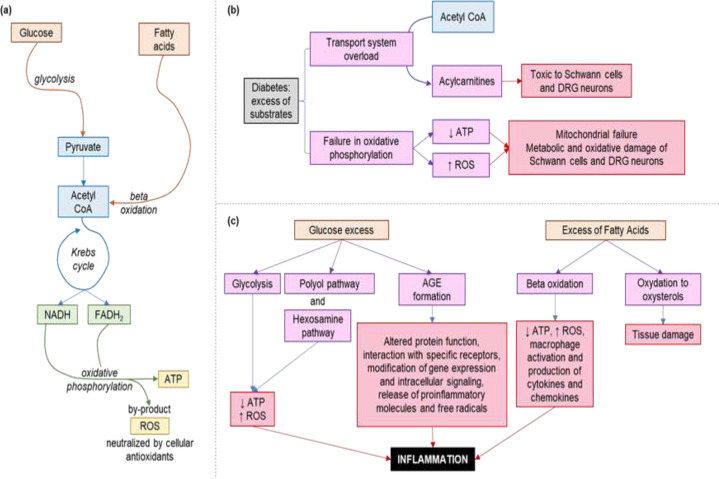
Relationship of the glucose and fatty acid metabolism with DN. (a) The normal metabolism of glucose and fatty acids, which, through a series of complementary mechanisms, end in the production of ATP. (b) The effects of DM on the normal metabolism of glucose and fatty acids, overloading the transport system and causing failure of oxidative phosphorylation. (c) The effects of an excess of glucose and fatty acids and their relation to inflammation and tissue damage. (34)

**Role of dysfunction in the microcirculation**

Poor blood supply to peripheral nerves is another possible pathological mechanism involved in DN. Microcirculatory dysfunction is associated with peripheral nerve dysfunction, leading to further nerve damage. DM patients exhibit higher capillary density in the endometrium, probably influenced by DM-induced nerve ischemia (60). The basement membrane of blood vessels undergoes thickening and is also associated with nerve damage (61). DM also decreases vascular formation mediators such as insulin-like growth factors, vascular endothelial growth factor (VEGF), nerve growth factor (NGF), and angiopoietins (62).

**Role of insulin resistance**

Normally, insulin acts as a growth factor for sensory neurons, allowing the growth of neuronal processes. Insulin receptors are expressed in the sensory neurons of the DRGs and axons, especially in the nodes of Ranvier (63, 64). Glycemic control with insulin has little effect on neuropathy in patients with T2DM given the insulin resistance developed by the neurons, similar to that of the muscle and adipose tissue. In contrast, in T1DM, the benefit on neuropathy is notorious (65, 66).

**Mechanisms of neuropathic pain**

Nearly half of DN patients develop neuropathic pain, frequently manifested as a spontaneous burning pain in the feet (67). Allodynia, paresthesia, and loss of sensitivity have also been reported. The presence or absence of neuropathic pain is probably due to a complex interaction of genetic factors, somatosensory circuitry, and psychological factors (68).

 Being female increases the risk of painful DN. The severity of the neuropathy, poor glycemic control, renal dysfunction, and a higher body mass index are risk factors for neuropathic pain and are associated with neuropathy progression (69, 70).

Damaged sensory neurons present hyperexcitability, and generate spontaneous activity by action potentials in the absence of a stimulus and an altered response. This aberrant activity is what initiates and allows the continuation of neuropathic pain (71, 72). The pathogenesis of neuropathic pain also involves genetic variations of ion channels and alterations in their expression, trafficking, and post-translational modifications, such as an increased expression of the Nav1.8 subunit of the channel, voltage-gated sodium in sensory neurons, which contributes to hyperexcitability of DRG neurons (73-75).

 In addition, hyperglycemia increases the expression of glyoxal, a reactive metabolite that forms PFGA by modifying ion channel cellular proteins, altering their function (76.^77^). In DN, there is an increased influx of spontaneously active nociceptors into the spinal cord, which enhances synaptic transmission, further amplifying nociceptive signaling in a process called central sensitization. Microglia exhibit a proinflammatory phenotype, although the exact form is unknown. These cells can release factors, such as brain-derived neurotrophic factor, that amplify nociceptive synaptic signaling within the spinal cord and contribute to mechanical pain-related hypersensitivity (78.^79^).

**Sensory-motor neuropathy**

Several clinical syndromes of DN have been described, very different from each other, although often coexisting. Sensorimotor neuropathy includes distal sensory polyneuropathy, acute mononeuropathies, multiple mononeuropathies, and radiculopathies (80). Distal sensory polyneuropathy is the most frequent form and in more than 50% of cases, it generates symptoms like burning pain, electrical or stabbing sensations, paresthesia, and hyperesthesia, which are usually worse at night and reduce the ability to perform daily activities (81, 82).

 In the examination of the lower extremities, loss of sensitivity to vibration, pressure pain, and temperature is usually found, as well as decreased or absent osteotendinous reflexes. When there is muscle weakness, it is usually mild (81, 83). Some patients develop early neuropathy, not necessarily associated with the use of insulin or oral hypoglycemic agents, and manifest only pain and paresthesia, which corresponds to another entity called insulin neuritis (84). In order of frequency, the most common acute mononeuropathies are paralysis of the oculomotor nerve and those of the trochlear and facial nerves (84). They are usually associated with nerve ischemia and generally appear during a transition period in DM, such as after an episode of hyperglycemia or hypoglycemia, when the insulin therapy regimen is started or adjusted, or when there is rapid weight loss (83).

 Multiple mononeuropathies are unilateral or asymmetric painful neuropathies that start abruptly in one nerve and then sequentially or irregularly affect other nerves (80). These radicular-plexopathies present sub-acutely with pain followed by weakness, and mainly affect older people with mild or undiagnosed DM. The main forms are cervical, thoracic, and lumbosacral radicular-plexopathy, and they can present separately or simultaneously (85). Of these, lumbosacral radicular-plexopathy is the one that produces the greatest morbidity, with intense pain that begins in the waist or hips and extends to the thigh and knee on one side, associated with kneeling and might be more intense at night. Pain is the main discomfort at the beginning, but gradually weakness and atrophy become the biggest problem, mainly affecting the pelvic girdle and the thighs, and progressively it may spread more distally. Due to the above, it is also known as diabetic amyotrophy (83–85).

**Autonomic neuropathies**

Autonomic neuropathies can affect cholinergic, adrenergic, and peptidergic fibers, and can be sub-clinically detected with tests or become evident with signs and symptoms (86).

Cardiovascular autonomic neuropathy arises as a result of the interaction between glycemic control, duration of DM, age-related neuronal wear, and blood pressure (86). Its manifestations include altered heart rate variability, tachycardia at rest, exercise intolerance, blood pressure dysregulation, and orthostatic hypotension (87, 88). Although symptoms appear years after DM onset, subclinical involvement can be detected as early as 1 year after T2DM diagnosis and 2 years after T1DM diagnosis. This form of neuropathy is associated with the highest morbidity and mortality (88, 89). 

Gastrointestinal autonomic neuropathy is usually a diagnosis of exclusion due to the difficulty in evaluating gastrointestinal function in humans. It affects up to 75% of people with DM (87, 88). It produces nausea, bloating, abdominal pain, diarrhea, constipation, and delayed gastric emptying, altering the absorption of medications, hindering glycemic control, and producing malnutrition and a poor quality of life (87, 90). Another type of autonomic neuropathy is erectile dysfunction, which affects more than 50% of men with DM and is caused by neuropathy and endothelial dysfunction. Given its close relationship with endothelial dysfunction, erectile dysfunction is an early marker of cardiovascular risk (91-93). Bladder dysfunction occurs due to involvement of autonomic and sensory nerve fibers (94, 95). The first thing that is altered is the sensitivity in the bladder, producing delayed urinary reflexes, increased bladder capacity, and urinary retention (88, 95). This can happen asymptomatically, becoming the dysfunction evident when a urinary tract infection occurs secondary to the increase in residual urine volume (96). Besides, autonomic neuropathy initially produces loss of thermoregulatory sweating in the extremities and anterior abdomen, culminating in global anhidrosis. In some cases, it causes hyperhidrosis (97). 

**Diagnosis**

DN presents in a heterogeneous manner and on many occasions without a correlation with glycemia figures, possibly, being it not impossible to go together with renal DM complications, which lowers the blood sugar level. So, defining it accurately and, even more, classifying it is sometimes controversial. For a better understanding of its manifestations, it is usually classified into two groups, being generalized symmetrical polyneuropathy which includes the acute sensory, chronic sensory-motor, and autonomic form, and the focal and multifocal neuropathy. The last group includes the list of cranial, truncal, focalized in one extremity, proximal motor or neuropathy amyotrophic, and chronic inflammatory demyelinating polyneuropathy forms (80, 81, 98). The diagnosis of DN is clinical, based on an adequate anamnesis and physical examination, restricting objective confirmatory tests mainly to the field of research or in the case of atypical clinical presentations (10.88). The heterogeneous presentations like tingling, together with lancinating pain, accompanied by weakness and instability in the extremities, from distal to proximal onset, occur in most patients and are the necessary elements for diagnosis (10, 80, 88). The signs and symptoms (99) together with the systemic manifestations (81) are summarized in Table 1, which are easy to identify in a clinical evaluation. 

The great variety of signs and symptoms can be organized into a series of scales that can help us to formalize the diagnosis. Despite the passage of time, the Toronto Clinical Neuropathy Score is still used due to its easy application and high reproducibility in any medium. This scale considers the symptoms of foot pain, numbness, tingling, weakness, ataxia, and upper limb symptoms, as well as knee and ankle reflexes, finishing with the sensory tests using a monofilament, temperature perception, vibration, position, and light touch. It offers a total score from 0 to 19, where a higher score reflects greater nerve damage (100,101). If a patient with pain, paresthesia, and/or weakness complains of atypical sub-acutely or acutely characteristics, or presents a heterogeneous distribution, an objective diagnostic laboratory test is necessary (99,102) to rule out vitamin B12 deficiency, especially if the patient is a long-term metformin user (103), as well as thyroid function control, even screening for autoimmune diseases. Rarely, a biopsy of the sural or radial nerve is necessary (10, 98, 104). 

**Table 1:** Common systemic signs and symptoms of Diabetic neuropathy

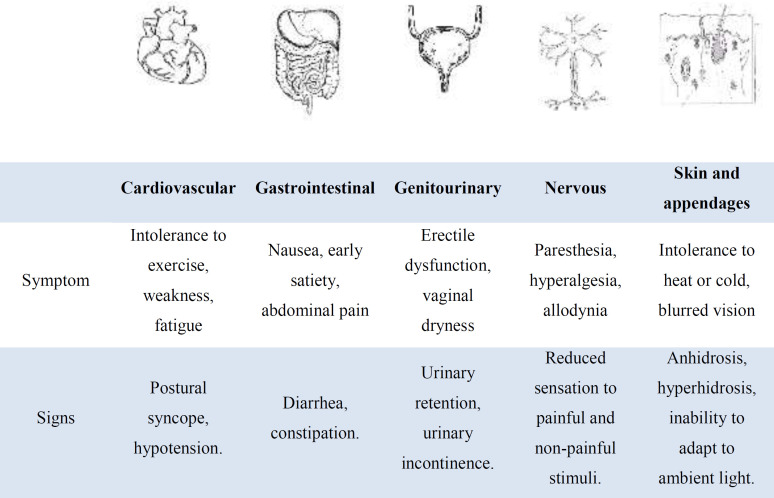

**Differential diagnosis**

As has been noted, there are different forms of presentation of DN, with distal-onset generalized symmetric polyneuropathy being the most frequent form. However, it must be taken into account that not all diabetic patients have neuropathy secondary to DM. In fact, in approximately 1 in 10 diabetics with neuropathy, DM is not the responsible entity, but rather some other cause is involved (34,105).

That is why it is necessary to assess other etiologies that can generate neuropathy such as alcohol abuse, genetic alterations, neoplasms, medications like chemotherapy and HIV treatments, and amyloidosis. An adequate clinical history is essential to know what type of patient should have a more exhaustive search for another cause. (10.34)

**Screening**

All screening tests must be easy and fast to apply, and their results reliable, so the arsenal of tests used for DN screening is very small. The 10-gram monofilament together with the 128 Hz tuning fork have similar screening power, compared with other more expensive and difficult-to-apply methods to discriminate DN (10,34,105–107). The American Diabetes Association, as well as the American Academy of Neurology, recommend screening for ND at diagnosis and annually for patients with T2DM, and 5 years after diagnosis and annually thereafter for patients with T1DM (10,105).

**Prevention**

In both T1DM and T2DM, the base harm is hyperglycemia, so evidently, glycemic control is the base of DN prevention, however, the outcome does not seem to be the same in the different types of DM, since the incidence decreases very slightly in patients with T2DM compared to patients with T1DM, making us know that the pathophysiological mechanism of each type of DM plays a crucial role in this complication (107,108). In not many years we will be able to have adequate studies at hand to take a position on other types of interventions such as metabolic surgeries, which help in the remission of DM2, but we still cannot adequately distinguish what role they play in complications such as DN.

**Management**

The management of DN includes 4 main strategies: glycemic control, intervention in diet and lifestyles, therapies aimed at the pathogenesis of the disease, and symptomatic relief of neuropathic pain (109). The latter being perhaps the main objective sought by the patient but which the clinician must always consider within the global management mentioned.

**Glycemic control**

The DCCT study showed that intensive glycemic control can delay the onset of DN in patients with T1DM. After 6.5 years of follow-up, the intensive glycemic control group with a mean HbA1c of 7.4% had an increase in frequency of DN from 7% to 9% compared to the conventional glycemic control group with a mean HbA1c of 9.1% what presented an increase from 5% to 17% in the frequency of DN (110). Glycemic control is less effective in preventing the progression of DN in patients with DM2. The UKPDS study did not show a significant difference in the frequency of DN between the conventional and intensive glycemic control groups with sulfonylureas or insulin (111). These data should not lead us to suppose that glycemic control is less relevant in T2DM as it affects also other organs and physiological pathways, such as the nervous system, and must be controlled (109, 112). The previously mentioned studies involve a large international population and assessed the intensity of glycemic control by a trimestral HbA1c, which proved to be an important tool in disease management. The result of the HbA1c value has to be considered not to be completely adequate in patients with hemoglobin alterations, like in any anemia, iron deficiencies, chronic kidney disease, polycythemia, and related clinical conditions. In addition, the high glycemic variability that a patient with frequent dietary transgressions can present can only be distinguished in daily continuous glucose monitoring. This is why numerous studies have appeared in recent years that attempt to assess whether glycemic variability plays a role in the progression of complications, finding that it does play a role as an independent factor in the development and progression of DN in T1DM as well in T2DM; therefore, the use of continuous glycemic monitoring systems may become more relevant (113–116). *** The most recent studies have been summarized in Table 2.

*** References 117–123 are in Table 2.

**Diet and lifestyle**

Evaluating the impact of diet itself in the treatment of DN, we have not found strong evidence of direct benefit, however, it is deduced that an adequate diet that allows glycemic control should have a positive impact on the complications of DN, so on ND. Within the range of possibilities, the total vegetarian or vegan diet can have a beneficial effect on the symptomatic control of DN (124). Another option that has shown a benefit in the control of metabolic diseases is the Mediterranean diet, having as its main characteristic the high consumption of fats from vegetable oils and proteins from fish meat. This has not generated direct evidence so far in the improvement of DN symptoms, but in the progression of the disease and in inflammatory mechanisms that are related to the pain pathways, which can reduce symptoms this way (125-127). Very low-calorie diets, between 200-500 kcal/day, could have a beneficial effect by reducing insulin resistance and improving glycemic control figures, but given the possibility of an increase in ketone bodies, there is no clear picture(128). Nutrient deficiencies might be an additional risk in time. Obesity control and weight reduction to a normal MIC can improve the structure and function of peripheral nerves (129). 

Metabolic surgery in DM to generate greater and faster weight loss is approved by health securities in some countries.

Ketogenic diets and Dietary-Approaches-to-Stop-Hypertension (DASH) diets have only shown clear benefits in the management of arterial hypertension. 

Mild to moderate-intense physical exercise can have a beneficial impact on neuropathic pain reduction and DN control (130) not only because of the control effect on hyperglycemia itself but also because it influences plasticity mechanisms that can improve symptoms (131).

**Disease-modifying therapies**

Two drugs that are approved for the treatment of DN in multiple countries, stand out: alpha-lipoic acid, which reduces oxidative stress, and benfotiamine, which inhibits the accumulation of PFGA (132,133). 

Several randomized clinical trials have shown the efficacy and safety of alpha-lipoic acid 600 mg daily in reducing neuropathic symptoms, intravenously after 3 weeks and orally after 5 weeks of treatment (134–137). Benfotiamine is a pro-drug of thiamin (vitamin B1). The BENDIP (Benfotiamine in Diabetic Polyneuropathy) clinical trial showed a significant improvement in neuropathic symptoms after 6 weeks of treatment with a dose of 300 mg every 12 hours, which proved to be the optimal effective and safe dose (138).

More large-scale studies are needed before these drugs can be recommended in the clinical practice.

**Table 2:** Impact of the glycemic variability on Diabetic neuropathy

**Study**	**Populación and type**	**Result**
Oyibo SO et al. 2002. (117)	Randomized clinical trial in 20 T1DM patients.	It was found that the mean glycemic variability was higher in painful DN, but without statistical significance.
Kwai NC et al. 2016. (118)	Randomized clinical trial in 17 T1DM patients.	In patients with higher glycemic variability, greater neuronal excitability was found.
Akaza M et al. 2018. (119)	Randomized clinical trials in 13 and 27 T1DM and T2DM patients, respectively.	Glycemic variability was independently associated with an increased risk of medial plantar neuropathy.
Dahlin LB et al. 2020. (120)	Randomized clinical trial in 159 T1DM patients.	The perception of vibrations measured in Hertz was evaluated, finding fewer vibrations in patients with better glycemic control.
Mizokami-Stout KR et al. 2020. (121)	Retrospective cohort in 5936 T1DM patients.	A significant association of painful DN was found with those patients who presented acute diabetic complications during follow-up.
Feng ZQ et al. 2021. (122)	Randomized clinical trial in 95 T1DM patients.	A significant correlation was found between time in the glycemic range and HbA1c-independent sudomotor dysfunction.
Yang J et al. 2021. (123)	Cross-sectional study in 364 T2DM patients.	Time in range correlated with painful DN and stands out as a valuable clinical assessment measure.

**Pain management**

Pain relief in patients with DN has been a great challenge and its adequate approach must take into account multiple factors. Despite the high prevalence of DN, only 40% of patients receive a treatment considered first or second line in the treatment of DN. A similar percentage does not receive any treatment (139). There are more than 30 drugs recommended for the treatment of DN, with different efficacy, safety, and level of evidence. Therapeutic efficacy is considered with a significant reduction in symptoms, approximately 50% on pain scales. But none of them has been shown to achieve complete pain relief. In clinical practice, two approaches to pain management stand out. The first focused on the DN phenotype and neuropathic pain and the second approach focused on the presence of comorbidities, including anxiety, depression, sleep disorders, chronic complications of DM, cardiovascular diseases, among others(140).

Two DN phenotypes have been described: a paresthesia-like pain phenotype and a paroxysmal pain phenotype. For the paresthesia-like pain phenotype, it is recommended to start treatment with duloxetine (141). While for the phenotype of pain with paroxysms, treatment with high doses of pregabaline is recommended (142). Both drugs are recognized as first-line drugs in DN. Regarding the approach according to comorbidities, the use of duloxetine is recommended in patients with depression. In patients with anxiety or sleep disorders, it is preferred to initiate treatment with pregabalin. In the absence of improvement, tricyclic antidepressants could be added as second-line therapy (133), always emphasizing to the patient that the symptomatic relief of pain is not instantaneous and that the dose should be titrated according to each patient and based on the doses accepted until now. The drugs are described in table 3 with the recommended dose, additionally, the time in weeks on average that it must have been administrated to show an effect (112). *** Finally, the treatment must be evaluated and valued by the patient, currently existing various scales such as the Diabetes Quality of Life (DQOL) or the Diabetes-Specific Quality of Life Scale (DSQOLS) (151), which attempt to assess objectively the improvement or deterioration in the quality of life (152).

*** References 143–150 are in Table 2.

**Table 3:** Drugs involved in the treatment of Diabetic neuropathy

**Drug**	**Intervention**	**Goal**	**Dose (mg/d)**	**Effect (weeks)**
Duloxetine (143)	First-line Anti-depressive	SNRIs	40-60	10-12
Pregabalin (144)	First-line Anti-convulsant	α2-δ ligand	300-600	8-12
Alfa-lipoic acid (145)	First-line Neuromodulator	NF-κB, ROS, TRPV1	600-1800	4-6
Benfotiamine (146)	First-line Neuromodulator	Hexosamine pathway	100-300	4-6
Amitriptyline (147)	Second-line Anti-depressant	SNRIs	25-100	2-4
Valproic acid (148)	Second-line Anticonvulsant	GABA	1000-1200	4-12
Gabapentin (149)	Second-line Anti-convulsant	GABA	900-3600	4-6
Carbamazepine (150)	Second-line	Sodium channels	400-800	8-12

GABA: gamma-aminobutyric acid; NF-κB: Nuclear factor kappa B; ROS: reactive oxygen species; SNRI: Serotonin–norepinephrine reuptake inhibitor; TRPV1: transient receptor potential vanilloid 1

**Therapies under study**

There is a lot of interest in therapeutic agents focused on other points within the pathophysiology of DN, which is why new drugs are under study (153,154). Desensitization of the temperature-sensitive transient receptor potential channel in nociceptive neurons has been proposed as an interesting therapeutic option considering the pain pathways, through the application of topical capsaicin, which is still only approved for the management of painful DN in feet (155). The use of low-voltage electrical current stimulation of the spinal cord with success in some studies is also under investigation (156,157).

 Mutations in voltage-gated sodium channels such as Nav1.7 have been implicated in painful DN, and are the target of antagonists such as the drug Xenon402, which is useful in erythromelalgia and has the potential to be used in other types of neuropathic pain (158,159). The intrathecal administration of drugs such as morphine and ziconotide allows direct release into the cerebrospinal fluid, with fewer side effects than systemic administration; however, their use in DN has not been evaluated, and this could be complicated by the difficulty in healing of wounds that people with DM are prone to (160,161).

 The benefit of topical application of O2 and CO2 nanobubbles for the treatment of DN symptoms is also being studied in more than 50% of patients with success so far (162). In conclusion, Diabetes mellitus causes damage to peripheral nerves, with a variety of clinical manifestations. The most common form is distal symmetric polyneuropathy. Control of glycemia, blood pressure, and other components of the metabolic syndrome contribute to prevent or postpone it, as in other cases, stopping the progression of this condition or in the worse cases, reducing its severity. Intervention in lifestyles such as an appropriate diet and exercise also provide benefits in its management. 

Pharmacological interventions may be aimed at relieving pain, but there is considerable progress made in disease-modifying therapies. There are many aspects of this disease that are still being investigated to improve its prevention and treatment.

## References

[1] Skljarevski V, Lledo A (2006). Diabetic Neuropathies. Arch Neurol.

[2] Boulton AJM (2014). Diabetic neuropathy and foot complications. Handb Clin Neurol.

[3] Schwarz PEH, Reimann M, Li J, et al (2007). The Metabolic Syndrome - a global challenge for prevention. Horm Metab Res.

[4] Standl E, Khunti K, Hansen TB, Schnell O (2019). The global epidemics of diabetes in the 21st century: Current situation and perspectives. Eur J Prev Cardiol.

[5] Zimmet P, Alberti KG, Shaw J (2001). Global and societal implications of the diabetes epidemic. Nature.

[6] American Diabetes Association Professional Practice Committee (2021). Improving care and promoting health in populations: standards of medical care in diabetes-2022. Diabetes Care.

[7] American Diabetes Association Professional Practice Committee 3 (2021). Prevention or delay of type 2 diabetes and associated comorbidities: standards of medical care in diabetes-2022. Diabetes Care.

[8] Rodríguez Bolaños R de los Á, Reynales Shigematsu LM, Jiménez Ruíz JA, Juárez Márquezy SA, Hernández Ávila M (2010). Direct costs of medical care for patients with type 2 diabetes mellitus in Mexico micro-costing analysis. Rev Panam Salud Publica December.

[9] Gordois A, Scuffham P, Shearer A, Oglesby A, Tobian JA (2003). The health care costs of diabetic peripheral neuropathy in the US. Diabetes Care.

[10] Draznin B, Aroda VR, American Diabetes Association Professional Practice Committee (2022). 12. Retinopathy, neuropathy, and foot care: standards of medical care in diabetes-2022. Diabetes Care.

[11] Kiyani M, Yang Z, Charalambous LT, et al (2020). Painful diabetic peripheral neuropathy: Health care costs and complications from 2010 to 2015. Neurol Clin Pract.

[12] Callaghan BC, Kerber KA, Lisabeth LL, et al (2014). Role of neurologists and diagnostic tests on the management of distal symmetric polyneuropathy. JAMA Neurol.

[13] Candrilli SD, Davis KL, Kan HJ, Lucero MA, Rousculp MD (2007). Prevalence and the associated burden of illness of symptoms of diabetic peripheral neuropathy and diabetic retinopathy. J Diabetes Complications.

[14] Adler AI, Boyko EJ, Ahroni JH (1997). Risk factors for diabetic peripheral sensory neuropathy Results of the Seattle Prospective Diabetic Foot Study. Diabetes Care.

[15] Pirart J (1977). Diabetes mellitus and its degenerative complications: a prospective study of 4,400 patients observed between 1947 and 1973 (author’s transl). Diabetes Metab.

[16] Edwards JL, Vincent AM, Cheng HT, Feldman EL (2008). Diabetic neuropathy: Mechanisms to management. Pharmacol Ther.

[17] Ang L, Jaiswal M, Martin C, Pop-Busui R (2014). Glucose control and diabetic neuropathy: lessons from recent large clinical trials. Curr Diab Rep.

[18] Martin CL, Albers JW, Pop-Busui R (2014). Neuropathy and related findings in the diabetes control and complications trial/epidemiology of diabetes interventions and complications study. Diabetes Care.

[19] Franklin GM, Kahn LB, Baxter J, Marshall JA, Hamman RF (1990). Sensory neuropathy in non-insulin-dependent diabetes mellitus The San Luis Valley Diabetes Study. Am J Epidemiol.

[20] Partanen J, Niskanen L, Lehtinen J Natural history of peripheral neuropathy in patients with non-insulin-dependent diabetes mellitus. N Engl J Med1995.

[21] Dyck PJ, Kratz KM, Karnes JL, et al (1993). The prevalence by staged severity of various types of diabetic neuropathy, retinopathy, and nephropathy in a population-based cohort: the Rochester Diabetic Neuropathy Study. Neurology.

[22] Tesfaye S, Chaturvedi N, Eaton SEM, et al (2005). Vascular risk factors and diabetic neuropathy. N Engl J Med.

[23] Andersen ST, Witte DR, Dalsgaard EM, et al (2018). Risk factors for incident diabetic polyneuropathy in a cohort with screen-detected type 2 diabetes followed for 13 years: ADDITION-Denmark. Diabetes Care.

[24] Callaghan BC, Gao L, Li Y, et al (2018). Diabetes and obesity are the main metabolic drivers of peripheral neuropathy. Ann Clin Transl Neurol.

[25] Callaghan BC, Xia R, Banerjee M, et al (2016). Metabolic syndrome components are associated with symptomatic polyneuropathy independent of glycemic status. Diabetes Care.

[26] Callaghan BC, Xia R, Reynolds E (2016). Association between metabolic syndrome components and polyneuropathy in an obese population. JAMA Neurol.

[27] Hanewinckel R, Drenthen J, Ligthart S (2016). Metabolic syndrome is related to polyneuropathy and impaired peripheral nerve function: a prospective population-based cohort study. J Neurol Neurosurg Psychiatry.

[28] Lu B, Hu J, Wen J, et al (2013). Determination of peripheral neuropathy prevalence and associated factors in Chinese subjects with diabetes and pre-diabetes– ShangHai Diabetic neunopathy Epidemiology and Molecular Genetics Study (SH-DREAMS). PLoS One.

[29] Schlesinger S, Herder C, Kannenberg JM, et al (2019). General and abdominal obesity and incident distal sensorimotor polyneuropathy: insights into inflammatory biomarkers as potential mediators in the KORA F4/FF4 Cohort. Diabetes Care.

[30] Tesfaye S, Selvarajah D (2009). The Eurodiab study: what has this taught us about diabetic peripheral neuropathy?. Curr Diab Rep.

[31] Callaghan BC, Price RS, Feldman EL (2015). Distal Symmetric Polyneuropathy: A Review. JAMA.

[32] Prabodha LBL, Sirisena ND, Dissanayake VHW (2018). Susceptible and prognostic genetic factors associated with diabetic peripheral neuropathy: a comprehensive literature review. Int J Endocrinol.

[33] Baethge C, Goldbeck-Wood S, Mertens S (2019). SANRA-a scale for the quality assessment of narrative review articles. Res Integr Peer Rev.

[34] Feldman EL, Callaghan BC, Pop-Busui R, et al (2019). Diabetic neuropathy. Nat Rev Dis Primer.

[35] Gumy LF, Bampton ETW, Tolkovsky AM (2008). Hyperglycaemia inhibits Schwann cell proliferation and migration and restricts regeneration of axons and Schwann cells from adult murine DRG. Mol Cell Neurosci Febrero.

[36] Dunnigan SK, Ebadi H, Breiner A, et al (2013). Conduction slowing in diabetic sensorimotor polyneuropathy. Diabetes Care.

[37] Viader A, Sasaki Y, Kim S, et al (2013). Aberrant Schwann cell lipid metabolism linked to mitochondrial deficits leads to axon degeneration and neuropathy. Neuron.

[38] Callaghan BC, Gallagher G, Fridman V, Feldman EL (2020). Diabetic neuropathy: what does the future hold?. Diabetologia.

[39] Vincent AM, Calabek B, Roberts L, Feldman EL (2013). Biology of diabetic neuropathy. Handb Clin Neurol.

[40] Vincent AM, Callaghan BC, Smith AL, Feldman EL (2011). Diabetic neuropathy: cellular mechanisms as therapeutic targets. Nat Rev Neurol.

[41] Fernyhough P, McGavock J (2014). Mechanisms of disease: Mitochondrial dysfunction in sensory neuropathy and other complications in diabetes. Handb Clin Neurol.

[42] Fernyhough P (2015). Mitochondrial dysfunction in diabetic neuropathy: a series of unfortunate metabolic events. Curr Diab Rep.

[43] Chowdhury SKR, Smith DR, Fernyhough P (2013). The role of aberrant mitochondrial bioenergetics in diabetic neuropathy. Neurobiol Dis.

[44] Rumora AE, Lentz SI, Hinder LM (2018). Dyslipidemia impairs mitochondrial trafficking and function in sensory neurons. FASEB J.

[45] Feldman EL, Nave KA, Jensen TS, Bennett DLH (2017). New Horizons in diabetic neuropathy: mechanisms, bioenergetics, and pain. Neuron.

[46] Padilla A, Descorbeth M, Almeyda AL, Payne K, De Leon M (2011). Hyperglycemia magnifies Schwann cell dysfunction and cell death triggered by PA-induced lipotoxicity. Brain Res.

[47] Legrand-Poels S, Esser N, L’homme L (2014). Free fatty acids as modulators of the NLRP3 inflammasome in obesity/type 2 diabetes. Biochem Pharmacol.

[48] Singh VP, Bali A, Singh N, Jaggi AS (2014). Advanced glycation end products and diabetic complications. Korean J Physiol Pharmacol.

[49] Jang ER, Lee CS (2011). 7-ketocholesterol induces apoptosis in differentiated PC12 cells via reactive oxygen species-dependent activation of NF-κB and Akt pathways. Neurochem Int.

[50] Niki E Dual stressor effects of lipid oxidation and antioxidants. En: Oxidative Stress [Internet]. Elsevier.

[51] Mutemberezi V, Guillemot-Legris O, Muccioli GG (2016). Oxysterols: from cholesterol metabolites to key mediators. Prog Lipid Res.

[52] Vincent AM, Hayes JM, McLean LL (2009). Dyslipidemia-induced neuropathy in mice: the role of oxLDL/LOX-1. Diabetes.

[53] Nowicki M, Müller K, Serke H, et al (2010). Oxidized low-density lipoprotein (oxLDL)-induced cell death in dorsal root ganglion cell cultures depends not on the lectin-like oxLDL receptor-1 but on the toll-like receptor-4. J Neurosci Res.

[54] Vincent AM, Perrone L, Sullivan KA, et al (2007). Receptor for advanced glycation end products activation injures primary sensory neurons via oxidative stress. Endocrinology.

[55] Cotter MA, Cameron NE (2003). Effect of the NAD(P)H oxidase inhibitor, apocynin, on peripheral nerve perfusion and function in diabetic rats. Life Sci.

[56] Penno A, Reilly MM, Houlden H, et al (2010). Hereditary sensory neuropathy type 1 is caused by the accumulation of two neurotoxic sphingolipids. J Biol Chem.

[57] Zuellig RA, Hornemann T, Othman A, et al (2014). Deoxysphingolipids, novel biomarkers for type 2 diabetes, are cytotoxic for insulin-producing cells. Diabetes.

[58] Dohrn MF, Othman A, Hirshman SK, Bode H, Alecu I, Fähndrich E, et al (2015). Elevation of plasma 1-deoxy-sphingolipids in type 2 diabetes mellitus: a susceptibility to neuropathy?. Eur J Neurol.

[59] Hammad SM, Baker NL, El Abiad JM, et al (2017). Increased plasma levels of select deoxy-ceramide and ceramide species are associated with increased odds of diabetic neuropathy in type 1 diabetes: a pilot study. NeuroMolecular Med.

[60] Thrainsdottir S, Malik RA, Dahlin LB, et al (2003). Endoneurial capillary abnormalities presage deterioration of glucose tolerance and accompany peripheral neuropathy in man. Diabetes.

[61] Nowicki M, Kosacka J, Serke H, Blüher M, Spanel-Borowski K (2012). Altered sciatic nerve fiber morphology and endoneural microvessels in mouse models relevant for obesity, peripheral diabetic polyneuropathy, and the metabolic syndrome. J Neurosci Res.

[62] Schratzberger P, Walter DH, Rittig K, et al (2001). Reversal of experimental diabetic neuropathy by VEGF gene transfer. J Clin Invest.

[63] Frazier WA, Angeletti RH, Bradshaw RA (1972). Nerve Growth Factor and Insulin. Science.

[64] Fernyhough P, Willars GB, Lindsay RM, Tomlinson DR (1993). Insulin and insulin-like growth factor I enhance regeneration in cultured adult rat sensory neurones. Brain Res.

[65] Singh B, Xu Y, McLaughlin T, et al (2012). Resistance to trophic neurite outgrowth of sensory neurons exposed to insulin. J Neurochem.

[66] Kim B, McLean LL, Philip SS, Feldman EL (2011). Hyperinsulinemia induces insulin resistance in dorsal root ganglion neurons. Endocrinology.

[67] Abbott CA, Malik RA, van Ross ERE, Kulkarni J, Boulton AJM (2011). Prevalence and characteristics of painful diabetic neuropathy in a large community-based diabetic population in the U. K. Diabetes Care.

[68] von Hehn CA, Baron R, Woolf CJ (2012). Deconstructing the neuropathic pain phenotype to reveal neural mechanisms. Neuron.

[69] Raputova J, Srotova I, Vlckova E (2017). Sensory phenotype and risk factors for painful diabetic neuropathy: a cross-sectional observational study. Pain.

[70] Themistocleous AC, Ramirez JD, Shillo PR, et al (2016). The Pain in Neuropathy Study (PiNS): a cross-sectional observational study determining the somatosensory phenotype of painful and painless diabetic neuropathy. Pain.

[71] Suzuki Y, Sato J, Kawanishi M, Mizumura K (2002). Lowered response threshold and increased responsiveness to mechanical stimulation of cutaneous nociceptive fibers in streptozotocin-diabetic rat skin in vitro--correlates of mechanical allodynia and hyperalgesia observed in the early stage of diabetes. Neurosci Res.

[72] Ørstavik K, Namer B, Schmidt R, et al (2006). Abnormal function of C-fibers in patients with diabetic neuropathy. J Neurosci Off J Soc Neurosci.

[73] Bennett DLH, Woods CG (2014). Painful and painless channelopathies. Lancet Neurol.

[74] Blair NT, Bean BP (2002). Roles of tetrodotoxin (TTX)-sensitive Na+ current, TTX-resistant Na+ current, and Ca2+ current in the action potentials of nociceptive sensory neurons. J Neurosci Off J Soc Neurosci.

[75] Han C, Huang J, Waxman SG (2016). Sodium channel Nav1 8: Emerging links to human disease. Neurology.

[76] Bierhaus A, Fleming T, Stoyanov S, et al (2012). Methylglyoxal modification of Nav1 8 facilitates nociceptive neuron firing and causes hyperalgesia in diabetic neuropathy. Nat Med.

[77] Thornalley PJ, Langborg A, Minhas HS (1999). Formation of glyoxal, methylglyoxal and glyoxal 3-deoxyglucosone in the glycation of proteins by glucose. Biochem J.

[78] Woolf CJ (2011). Central sensitization: implications for the diagnosis and treatment of pain. Pain.

[79] Salter MW, Beggs S (2014). Sublime microglia: expanding roles for the guardians of the CNS. Cell.

[80] Deli G, Bosnyak E, Pusch G, Komoly S, Feher G (2013). Diabetic neuropathies: diagnosis and management. Neuroendocrinology.

[81] Boulton AJM, Vinik AI, Arezzo JC, et al (2005). Diabetic neuropathies: a statement by the American Diabetes Association. Diabetes Care.

[82] Tesfaye S, Selvarajah D (2012). Advances in the epidemiology, pathogenesis, and management of diabetic peripheral neuropathy. Diabetes Metab Res Rev.

[83] Ropper AH, Samuels MA, Klein JP, Prasad S (2019 ). Diseases of the peripheral nerves. Adams and victor’s principles of neurology [Internet].

[84] Bansal V, Kalita J, Misra UK (2006). Diabetic neuropathy. Postgrad Med J.

[85] Tracy JA, Dyck PJB (2008). The spectrum of diabetic neuropathies. Phys Med Rehabil Clin N Am.

[86] Spallone V, Bellavere F, Scionti L, et al (2011). Recommendations for the use of cardiovascular tests in diagnosing diabetic autonomic neuropathy. Nutr Metab Cardiovasc Dis.

[87] Chandrasekharan B, Srinivasan S (2007). Diabetes and the enteric nervous system. Neurogastroenterol Motil.

[88] Tesfaye S, Boulton AJM, Dyck PJ, et al (2010). Diabetic neuropathies: update on definitions, diagnostic criteria, estimation of severity, and treatments. Diabetes Care.

[89] Pop-Busui R (2010). Cardiac autonomic neuropathy in diabetes: a clinical perspective. Diabetes Care.

[90] Concepción Zavaleta MJ, Gonzáles Yovera JG, Moreno Marreros DM (2021). Diabetic gastroenteropathy: An underdiagnosed complication. World J Diabetes.

[91] Fan J, Peng T, Hui J (2021). Erectile dysfunction in type-2 diabetes mellitus patients: predictors of early detection and treatment. Urol Int.

[92] Kouidrat Y, Pizzol D, Cosco T, et al (2017). High prevalence of erectile dysfunction in diabetes: a systematic review and meta-analysis of 145 studies. Diabet Med J Br Diabet Assoc.

[93] Matsui H, Sopko NA, Hannan JL, Bivalacqua TJ (2015). Pathophysiology of erectile dysfunction. Curr Drug Targets.

[94] Liu G, Daneshgari F (2014). Diabetic bladder dysfunction. Chin Med J (Engl).

[95] Burakgazi AZ, Alsowaity B, Burakgazi ZA, Unal D, Kelly JJ (2012). Bladder dysfunction in peripheral neuropathies. Muscle Nerve.

[96] Gandhi J, Dagur G, Warren K, Smith NL, Khan SA (2017). Genitourinary complications of diabetes mellitus: an overview of pathogenesis, evaluation, and management. Curr Diabetes Rev.

[97] Freeman R (2005). Autonomic peripheral neuropathy. Lancet Lond Engl.

[98] Thomas PK (1997). Classification, differential diagnosis, and staging of diabetic peripheral neuropathy. Diabetes.

[99] Baron R, Binder A, Wasner G (2010). Neuropathic pain: diagnosis, pathophysiological mechanisms, and treatment. Lancet Neurol.

[100] Bril V, Tomioka S, Buchanan RA, Perkins BA (2009). Reliability and validity of the modified Toronto clinical neuropathy score in diabetic sensorimotor polyneuropathy. Diabet Med.

[101] Bril V, Perkins BA (2002). Validation of the Toronto clinical scoring system for diabetic polyneuropathy. Diabetes Care.

[102] Dyck PJ, Albers JW, Andersen H, et al (2011). Diabetic polyneuropathies: update on research definition, diagnostic criteria, and estimation of severity. Diabetes Metab Res Rev.

[103] Yang W, Cai X, Wu H, Ji L (2019). Associations between metformin use and vitamin B12 levels, anemia, and neuropathy in patients with diabetes: a meta-analysis. J Diabetes.

[104] Rota E, Quadri R, Fanti E, et al (2005). Electrophysiological findings of peripheral neuropathy in newly diagnosed type II diabetes mellitus. J Peripher Nerv Syst JPNS.

[105] Price R, Smith D, Franklin G, et al (2022). Oral and topical treatment of painful diabetic polyneuropathy: practice guideline update summary: report of the AAN guideline subcommittee. Neurology.

[106] Lehmann HC, Wunderlich G, Fink GR, Sommer C (2020). Diagnosis of peripheral neuropathy. Neurol Res Pract.

[107] Won JC, Park TS (2016). Recent advances in diagnostic strategies for diabetic peripheral neuropathy. Endocrinol Metab Seoul.

[108] Callaghan BC, Little AA, Feldman EL, Hughes RAC (2012). Enhanced glucose control for preventing and treating diabetic neuropathy. Cochrane Database Syst Rev.

[109] American Diabetes Association Professional Practice Committee (2022). Standards of medical care in diabetes. Diabetes Care.

[110] Diabetes Control, Complications Trial Research Group, Nathan DM, Genuth S, et al (1993). The effect of intensive treatment of diabetes on the development and progression of long-term complications in insulin-dependent diabetes mellitus. N Engl J Med.

[111] No authors listed (1998). Intensive blood-glucose control with sulphonylureas or insulin compared with conventional treatment and risk of complications in patients with type 2 diabetes (UKPDS 33). Lancet.

[112] Price R, Smith D, Franklin G, et al (2022). Oral and topical treatment of painful diabetic polyneuropathy: practice guideline update summary: report of the AAN guideline subcommittee. Neurology.

[113] Battelino T, Danne T, Bergenstal RM, et al (2019). Clinical targets for continuous glucose monitoring data interpretation: recommendations from the International Consensus on Time in Range. Diabetes Care.

[114] Beck RW, Connor CG, Mullen DM, Wesley DM, Bergenstal RM (2017). The fallacy of average: how using hba1c alone to assess glycemic control can be misleading. Diabetes Care.

[115] Danne T, Nimri R, Battelino T, et al (2017). International consensus on use of continuous glucose monitoring. Diabetes Care.

[116] Gouveri E, Papanas N (2022). The emerging role of continuous glucose monitoring in the management of diabetic peripheral neuropathy: a narrative review. Diabetes Ther.

[117] Oyibo SO, Prasad YDM, Jackson NJ, Jude EB, Boulton AJM (2002). The relationship between blood glucose excursions and painful diabetic peripheral neuropathy: a pilot study. Diabet Med J Br Diabet Assoc.

[118] Kwai NCG, Arnold R, Poynten AM, Krishnan AV (2016). Association between glycemic variability and peripheral nerve dysfunction in type 1 diabetes. Muscle Nerve.

[119] Akaza M, Akaza I, Kanouchi T (2018). Nerve conduction study of the association between glycemic variability and diabetes neuropathy. Diabetol Metab Syndr.

[120] Dahlin LB, Elgzyri T, Löndahl M, Ekman L, Lindholm E (2020). Improved metabolic control using glucose monitoring systems leads to improvement in vibration perception thresholds in type 1 diabetes patients. Acta Diabetol.

[121] Mizokami-Stout KR, Li Z, Foster NC, et al (2020). The contemporary prevalence of diabetic neuropathy in type 1 diabetes: findings From the T1D exchange. Diabetes Care.

[122] Feng ZQ, Guo QY, Wang W, et al (2021). Time in range, especially overnight time in range, is associated with sudomotor dysfunction in patients with type 1 diabetes. Diabetol Metab Syndr.

[123] Yang J, Yang X, Zhao D (2021). Association of time in range, as assessed by continuous glucose monitoring, with painful diabetic polyneuropathy. J Diabetes Investig.

[124] Crane MG, Sample C (1994). Regression of diabetic neuropathy with total vegetarian (Vegan) diet. J Nutr Med.

[125] Hernández Ruiz de Eguilaz M, Batlle MA, Martínez de Morentin B, et al (2016). Alimentary and lifestyle changes as a strategy in the prevention of metabolic syndrome and diabetes mellitus type 2: milestones and perspectives. A Sist Sanit Navar.

[126] Esposito K, Maiorino MI, Bellastella G (2015). A journey into a Mediterranean diet and type 2 diabetes: a systematic review with meta-analyses. BMJ Open.

[127] Estruch R, Martínez-González MA, Corella D, et al (2006). Effects of a Mediterranean-style diet on cardiovascular risk factors: a randomized trial. Ann Intern Med.

[128] Michalsen A (2010). Prolonged Fasting as a Method of Mood Enhancement in Chronic Pain Syndromes: A Review of Clinical Evidence and Mechanisms. Curr Pain Headache Rep.

[129] Bönhof GJ, Herder C, Strom A (2019). Emerging biomarkers, tools, and treatments for diabetic polyneuropathy. Endocr Rev.

[130] Kluding PM, Pasnoor M, Singh R, et al (2012). The effect of exercise on neuropathic symptoms, nerve function, and cutaneous innervation in people with diabetic peripheral neuropathy. J Diabetes Complications.

[131] Rodríguez JH, Puig MEL (2010). Role of physical exercise in persons presenting with diabetes mellitus. Revista Cubana de Endocrinología.

[132] Bondar A, Popa AR, Papanas N, et al (2021). Diabetic neuropathy: A narrative review of risk factors, classification, screening and current pathogenic treatment options (Review). Exp Ther Med.

[133] Ardeleanu V, Toma A, Pafili K, et al (2020). Current pharmacological treatment of painful diabetic neuropathy: a narrative review. Medicina (Kaunas).

[134] Javed S, Petropoulos IN, Alam U, Malik RA (2015). Treatment of painful diabetic neuropathy. Ther Adv Chronic Dis.

[135] Ziegler D (2008). Painful diabetic neuropathy: treatment and future aspects. Diabetes Metab Res Rev.

[136] Ziegler D, Nowak H, Kempler P, Vargha P, Low PA (2004). Treatment of symptomatic diabetic polyneuropathy with the antioxidant alpha-lipoic acid: a meta-analysis. Diabet Med J Br Diabet Assoc.

[137] Papanas N, Ziegler D (2014). Efficacy of α-lipoic acid in diabetic neuropathy. Expert Opin Pharmacother.

[138] Stracke H, Gaus W, Achenbach U, Federlin K, Bretzel RG (2008). Benfotiamine in diabetic polyneuropathy (BENDIP): results of a randomized, double-blind, placebo-controlled clinical study. Exp Clin Endocrinol Diabetes.

[139] Alam U (2020). Diabetic neuropathy collection: treatment of diabetic neuropathy. Diabetes Ther.

[140] Rolim LC, Koga da Silva EM, De Sá JR, Dib SA (2017). A systematic review of treatment of painful diabetic neuropathy by pain phenotype versus treatment based on medical comorbidities. Front Neurol.

[141] Bouhassira D, Wilhelm S, Schacht A, et al (2014). Neuropathic pain phenotyping as a predictor of treatment response in painful diabetic neuropathy: data from the randomized, double-blind, COMBO-DN study. Pain.

[142] Holbech JV, Bach FW, Finnerup NB (2015). Imipramine and pregabalin combination for painful polyneuropathy: a randomized controlled trial. Pain.

[143] Hossain SM, Hussain SM, Ekram ARMS (2016). Duloxetine in Painful Diabetic Neuropathy: A Systematic Review. Clin J Pain.

[144] Arezzo JC, Rosenstock J, Lamoreaux L, Pauer L (2008). Efficacy and safety of pregabalin 600 mg/d for treating painful diabetic peripheral neuropathy: a double-blind placebo-controlled trial. BMC Neurol.

[145] Mijnhout GS, Alkhalaf A, Kleefstra N, Bilo HJG (2010). Alpha-lipoic acid: a new treatment for neuropathic pain in patients with diabetes?. Neth J Med.

[146] Popa A, Bungau S, Cosmin Mihai V, et al (2019). Evaluating the efficacy of the treatment with benfotiamine and alpha-lipoic acid in distal symmetric painful diabetic polyneuropathy. Rev Chim -Buchar.

[147] Bansal D, Bhansali A, Hota D, Chakrabarti A, Dutta P (2009). Amitriptyline vs. pregabalin in painful diabetic neuropathy: a randomized double-blind clinical trial. Diabet Med J Br Diabet Assoc.

[148] Kochar DK, Jain N, Agarwal RP (2002). Sodium valproate in the management of painful neuropathy in type 2 diabetes- a randomized placebo-controlled study. Acta Neurol Scand.

[149] Majdinasab N, Kaveyani H, Azizi M (2019). A comparative double-blind randomized study on the effectiveness of Duloxetine and Gabapentin on painful diabetic peripheral polyneuropathy. Drug Des Devel Ther.

[150] Saeed T, Nasrullah M, Ghafoor A, Shahid R (2014). Efficacy and tolerability of carbamazepine for the treatment of painful diabetic neuropathy in adults: a 12-week, open-label, multicenter study. Int J Gen Med.

[151] Van Acker K, Bouhassira D, De Bacquer D, et al (2009). Prevalence and impact on quality of life of peripheral neuropathy with or without neuropathic pain in type 1 and type 2 diabetic patients attending hospital outpatients clinics. Diabetes Metab.

[152] Trikkalinou A, Papazafiropoulou AK, Melidonis A (2017). Type 2 diabetes and quality of life. World J Diabetes.

[153] Rastogi A, Jude EB (2021). Novel treatment modalities for painful diabetic neuropathy. Diabetes Metab Syndr.

[154] Qureshi Z, Ali MN, Khalid M (2022). An Insight into Potential Pharmacotherapeutic Agents for Painful Diabetic Neuropathy. J Diabetes Res.

[155] Brederson JD, Kym PR, Szallasi A (2013). Targeting TRP channels for pain relief. Eur J Pharmacol.

[156] de Vos CC, Meier K, Zaalberg PB, et al (2014). Spinal cord stimulation in patients with painful diabetic neuropathy: A multicentre randomized clinical trial. Pain.

[157] van Beek M, Geurts JW, Slangen R, et al (2017). Severity of neuropathy is associated with long-term spinal cord stimulation outcome in painful diabetic peripheral neuropathy: five-year follow-up of a prospective two-center clinical trial. Diabetes Care.

[158] Goldberg YP, Price N, Namdari R, et al (2012). Treatment of Na(v) 7-mediated pain in inherited erythromelalgia using a novel sodium channel blocker. Pain.

[159] McGowan E, Hoyt SB, Li X, Lyons KA, Abbadie C (2009). A peripherally acting nav1 7 sodium channel blocker reverses hyperalgesia and allodynia on rat models of inflammatory and neuropathic pain. Anesth Analg.

[160] Xing F, Yong RJ, Kaye AD, Urman RD (2018). Intrathecal Drug Delivery and Spinal Cord Stimulation for the Treatment of Cancer Pain. Curr Pain Headache Rep.

[161] Deer T, Rauck RL, Kim P, et al (2018). Effectiveness and safety of intrathecal ziconotide: interim analysis of the patient registry of intrathecal ziconotide management (PRIZM). Pain Pract Off J World Inst Pain.

[162] Lamour J, Grimm D, Smith E, Yaniv Z, Hurwitz P (2021). Treating diabetic peripheral neuropathy using a novel, nanotechnology-based topical formulation to improve pain, sensitivity, and function. Int J Diabetes Clin Res.

